# Molecular identification of *Wolbachia* and *Sodalis glossinidius* in the midgut of *Glossina fuscipes quanzensis* from the Democratic Republic of Congo

**DOI:** 10.1051/parasite/2019005

**Published:** 2019-02-07

**Authors:** Gustave Simo, Sartrien Tagueu Kanté, Joule Madinga, Ginette Kame, Oumarou Farikou, Gillon Ilombe, Anne Geiger, Pascal Lutumba, Flobert Njiokou

**Affiliations:** 1 Molecular Parasitology and Entomology Unit, Department of Biochemistry, Faculty of Science, University of Dschang PO Box 67 Dschang Cameroon; 2 Institute of Health and Society, Université Catholique de Louvain Clos Chapelle-aux-Champs 30 1200 Woluwe-Saint-Lambert Brussels Belgium; 3 Department of Biomedical Sciences, Institute of Tropical Medicine Nationalestraat 155 2000 Antwerp Belgium; 4 Department of Animal Biology and Physiology, Faculty of Science, University of Yaoundé I PO Box 812 Yaoundé Cameroon; 5 Mission Spéciale d’Eradication des Glossines Division Régionale Tsé-Tsé Adamaoua PO Box 263 Ngaoundéré Cameroon; 6 Institut national de recherche biomédicale Kinshasa Avenue de la démocratie N°5345 Gombe Kinshasa Democratic Republic of Congo; 7 UMR 177, IRD-CIRAD, CIRAD TA A-17/G, Campus International de Baillarguet Montpellier Cedex 5 France; 8 Center for Research on Filariasis and other Tropical Diseases (CRFILMT) PO Box 5797 Yaoundé Cameroon; 9 University of Yaoundé I, Faculty of Science PO Box 812 Yaoundé Cameroon; 10 Department of Tropical Medicine, University of Kinshasa B.P. 127 Kinshasa XI Democratic Republic of Congo

**Keywords:** *Glossina fuscipes quanzensis*, *Wolbachia*, *Sodalis glossinidius*, Democratic Republic of Congo, PCR

## Abstract

During the last 30 years, investigations on the microbiome of different tsetse species have generated substantial data on the bacterial flora of these cyclical vectors of African trypanosomes, with the overarching goal of improving the control of trypanosomiases. It is in this context that the presence of *Wolbachia* and *Sodalis glossinidius* was studied in wild populations of *Glossina fuscipes quanzensis* from the Democratic Republic of Congo. Tsetse flies were captured with pyramidal traps. Of the 700 *Glossina f. quanzensis* captured, 360 were dissected and their midguts collected and analyzed. *Sodalis glossinidius* and *Wolbachia* were identified by PCR. The *Wolbachia-*positive samples were genetically characterized with five molecular markers. PCR revealed 84.78% and 15.55% midguts infected by *Wolbachia* and *S. glossinidius,* respectively. The infection rates varied according to capture sites. Of the five molecular markers used to characterize *Wolbachia*, only the fructose bis-phosphate aldolase gene was amplified for about 60% of midguts previously found with *Wolbachia* infections. The sequencing results confirmed the presence of *Wolbachia* and revealed the presence of *S. glossinidius* in the midgut of *Glossina f*. *quanzensis.* A low level of midguts were naturally co-infected by both bacteria. The data generated in this study open a framework for investigations aimed at understanding the contribution of these symbiotic microorganisms to the vectorial competence of *Glossina fuscipes quanzensis*.

## Abstract (fr)

Au cours des 30 dernières années, les recherches sur le microbiome de différentes espèces de glossines ont généré des données substantielles sur la flore bactérienne de ces vecteurs cycliques des trypanosomes africains, l’objectif principal étant d’améliorer le contrôle des trypanosomiases. C’est dans cette optique que la présence de *Wolbachia* et *Sodalis glossinidius* a été étudiée dans des populations sauvages de *Glossina fuscipes quanzensis* de la République démocratique du Congo. Les glossines ont été capturées avec des pièges pyramidaux. Parmi les 700 *Glossina f. quanzensis* capturés, 360 ont été disséqués et leur estomac récupéré et analysé. *Sodalis glossinidius* et *Wolbachia* ont été identifiés par PCR. Les échantillons positifs pour *Wolbachia* ont été génétiquement caractérisés avec cinq marqueurs moléculaires. La PCR a révélé que 84,78 % et 15,55 % de l’intestin moyen étaient respectivement infectés par *Wolbachia* et *S. glossinidius*. Les taux d’infection variaient selon les sites de capture. Sur les cinq marqueurs moléculaires utilisés pour caractériser *Wolbachia*, seul le gène de la fructose bis-phosphate aldolase a été amplifié pour environ 60 % des intestins moyens précédemment identifiés porteurs de *Wolbachia*. Les résultats du séquençage ont confirmé la présence de *Wolbachia* et ont révélé la présence de *S. glossinidius* dans l’intestin moyen de *Glossina f. quanzensis*. Un faible taux des intestins moyens était naturellement co-infecté par les deux bactéries. Les données de cette étude ouvrent un cadre pour des recherches visant à comprendre la contribution de ces microorganismes symbiotiques sur la compétence vectorielle de *Glossina fuscipes quanzensis*.

## Introduction

Human African trypanosomiasis (HAT), also known as sleeping sickness, is caused by protozoan parasites of the genus *Trypanosoma*. The trypanosomes responsible for HAT belong to the *Trypanosoma brucei* species complex which is classically subdivided into three subspecies: *Trypanosoma brucei (T. b.) gambiense*, responsible for the chronic form of HAT in West and Central Africa, *T. b. rhodesiense*, responsible for the acute form in East and Southern Africa, and *T. b. brucei* which is pathogenic in animals but not in humans [28]. Apart from the latter subspecies, other pathogenic trypanosomes such as *Trypanosoma congolense* and *Trypanosoma vivax* cause African animal trypanosomiasis (AAT) or nagana.

Human African trypanosomiasis is an important public health problem in sub-Saharan Africa. On the basis of HAT-related mortality, HAT has been ranked ninth out of 25 human infectious and parasitic diseases in Africa [21, 47]. Efforts undertaken to control HAT during the last few decades brought the disease under control and led to its inclusion in the WHO “roadmap for eradication, elimination and control of neglected tropical diseases”, with a target set to eliminate HAT as a public health problem by 2020 [49]. To achieve this goal, sustainable control and surveillance measures must be developed to ensure complete interruption of disease transmission. In this light, alternative vector control using genetic engineering to generate insects capable of blocking the biological and cyclical transmission of the parasite is becoming an area of new investigation [41]. As a vector-borne disease, complete elimination of tsetse could stop the cyclical transmission of African trypanosomiases. Elimination has been achieved on Unguja Island in Zanzibar [44]; islands often have the advantage of limited tsetse species and also more importantly a lower chance of reinvasion. This seems unrealistic within mainland Africa where environmental and ecological conditions change considerably between biotopes and where several tsetse species coexist in the same biotope. In this context, the development of methods aimed at reducing vectorial competence of tsetse is becoming particularly important. Factors such as the tsetse species, the genetic variability within a given species, and the presence of symbiotic microorganisms seem to regulate vectorial competence of tsetse.

Tsetse flies harbor a variety of microorganisms including *Wigglesworthia glossinidia*, *Sodalis glossinidius* and *Wolbachia* spp. [3, 4, 23]. *Wigglesworthia glossinidia* is an obligate mutualist bacteria found in all tsetse species. It is essential for adult host fertility and for proper immune maturation [37, 46]. *Sodalis glossinidius* is a secondary symbiont which seems to promote midgut establishment of trypanosomes through complex biochemical mechanisms [11, 24, 48]. The third symbiont known as *Wolbachia* is trans-ovarially transmitted between different generations of tsetse flies. To enhance its transmission and its survival, *Wolbachia* has developed various mechanisms to alter host reproduction. One of its most common reproductive distortions is the cytoplasmic incompatibility that induces embryonic death due to disruptions in early fertilization events, as previously described in *G. morsitans morsitans* [6, 29]. Beside these well-known symbiotic microorganisms, other bacteria including *Bacillus subtilis*, *Serratia marcescens* and *Spiroplasma* [15, 33] were recently identified as new symbionts by DNA-based methods [15].

To better understand the biological role of symbiotic microorganisms in the vector competence of tsetse flies, investigations have been undertaken to improve our knowledge on the prevalence of *S. glossinidius* and *Wolbachia* in different tsetse species from various geographical areas [16, 20]. In tsetse of the *fuscipes* species, the first studies reported no *Wolbachia* infection, while other investigations revealed infections in some tsetse subspecies like *Glossina fuscipes fuscipes* [10, 16, 43]. Despite these contrasting results, no published data are available on the symbiotic microorganisms in *Glossina fuscipes quanzensis*. However, this tsetse species transmits African trypanosomes in the Republic of Congo and the Democratic Republic of Congo (DRC) where the highest number of HAT cases is reported [18]. In the current elimination context, investigations aiming to generate data on the infection rates of *S. glossinidius* and *Wolbachia* in *G. f. quanzensis* could provide baseline data to understand the vector competence of this tsetse species.

This study aimed to improve our knowledge on the symbiotic association between tsetse flies and its bacteria flora by using molecular tools to detect natural infections by *Wolbachia* and *S. glossinidius* in the midgut of *G. f. quanzensis* caught in the DRC.

## Materials and methods

### Study areas

**Figure 1:**
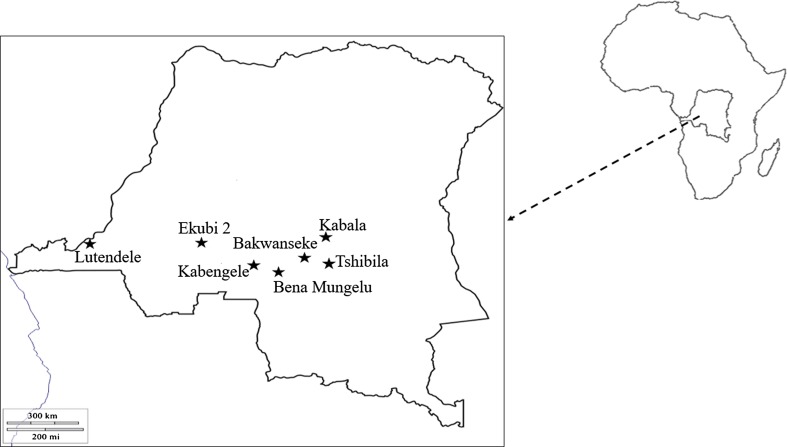
Map showing areas where tsetse flies were caught (stars).

Tsetse flies were captured in seven active HAT foci situated in three provinces (Bandundu, Eastern Kasaï and Kinshasa) of the DRC (Fig. 1).

In Bandundu province, tsetse flies were captured in Ekubi 2 (S04.55558°; E019.24772°), a village lying alongside the Kamtsa river which flows through a wide forest gallery. The vegetation of Ekubi 2 is predominantly characterized by bushy forest and the climate type is sub-equatorial. The primary means of subsistence for inhabitants is agriculture, fishing, fruit picking, hunting and small-scale animal husbandry.

In Eastern Kasaï province, tsetse flies were captured in five villages: Bena Mungelu (S06.03023°; E023.79057°), Kabengele (S06.03094°; E023.78581°), Kabala (S05.97876°; E023.76724°), Bakwanseke (S06.09524°; E023.82041°) and Tshibila (S06.06129°; E023.80552°). These villages share the same climatic, cultural and socio-economic characteristics. They lie in a shrubby savannah, with small streams that are all tributaries of the Lubilanji River. Farming and domestic livestock production represent the main economic activities for the inhabitants of these villages.

Kinshasa (S04.54011°; E019.22098°) is the capital of the DRC and lies along the Congo River. It is also the capital of “Kinshasa Province” with the urban area covering 4.5% of the 9965 km^2^ of the whole province. One part of the city lies on an alluvial plain up to the hills of the southern, western, and eastern sites. Kinshasa has a significant hydrographical network that crosses the mountains. In the periphery, the vegetation includes steppes, semi-deciduous and riverine forest islands, and wooded and grassy savannah [27]. It has an Atlantic littoral climate with low rainfall, low temperature, minimum sunshine, and maximum cloud mass. With a population of more than 8 million inhabitants, the association of unemployment and food precariousness has led many people to produce their own food in the suburban and rural areas of Kinshasa. Tsetse flies were captured in Lutendele, one of the suburban areas of Kinshasa.

### Trapping and dissection of tsetse flies

Two entomological surveys were undertaken in October 2009 and June 2010. During each survey, pyramidal traps [26] were set up for four consecutive days. The geographical coordinates of each trap were recorded using a global positioning system (GPS). Tsetse flies were collected once a day. All collected flies were morphologically identified, counted and sorted into teneral and non-teneral flies as described by Laveissière et al. [32]. Some of the live flies were randomly selected and dissected in a drop of 0.9% saline solution using a stereo-microscope. After dissection, the midgut of each fly was collected and preserved in a microtube containing 95° ethanol. Each microtube was kept at room temperature in the field, and later at −20 °C in the laboratory. Between two dissections, cross-contaminations were prevented by cleaning the dissecting instruments in a solution of sodium hydroxide (0.1 M) and then, in distilled water.

### DNA extraction

DNA was extracted from each tsetse fly midgut using the Cetyl trimethyl ammonium bromide (CTAB) method as described by Navajas et al. [36]. Briefly, the alcohol used to preserve midgut tissue was evaporated by incubating the opened microtube containing tsetse fly midgut at 80 °C in an oven for about one hour. Thereafter, midgut tissues were disrupted with a pestle in CTAB buffer (CTAB 2%; 1 M Tris, pH 8; 0.5 M EDTA, pH 8; 5 M NaCl). The disrupted tissues were incubated at 60 °C for 30 min before addition of chloroform/isoamylic alcohol mixture (24/1; V/V). The DNA was precipitated by addition of isopropanol (V/V) and centrifugation at 13,000 rpm for 15 min. The DNA pellets were washed twice with 70% cool ethanol and then dried at room temperature. The DNA pellets were finally re-suspended in 30 μL of sterile water and stored at −20 °C until use.

### Molecular identification of *S. glossinidius*

The presence of *S. glossinidius* was determined by amplifying a specific DNA fragment with pSG2 primers as described by Darby et al. [12]. Each PCR reaction was carried out in a DNA thermal cycler (TECHNE TC 4000). The amplification reactions were performed in a final volume of 15 μL containing 3 μL of DNA extract, 1.5 μL of 10X PCR reaction buffer, 2 mM of MgCl_2_, 20 picomoles of each primer (F1: 5′TGAAGTTGGGAATGTCG-3′ R1: 5′-AGTTGTAGCACAGCGTGTA-3′), 200 mM of each dNTP and 0.3 unit of Taq DNA polymerase. The amplification program began with a denaturation step at 94 °C for 3 min followed by 45 amplification cycles; each cycle containing a denaturation step at 94 °C for 30 s, an annealing step at 51 °C for 45 s, and an extension step at 72 °C for one minute followed by a final extension at 72 °C for 5 min.

After PCR amplification was completed, 10 μL of amplified products were analyzed by electrophoresis on a 2% agarose gel containing ethidium bromide. At the end of each electrophoresis, the gel was visualized under UV light and a picture of each gel was taken.

### Molecular identification of *Wolbachia*

The identification of *Wolbachia* was performed using primers pairs wsp F_1_ (GTCCAATARSTGATGARGAAAC) and wsp R_1_ (CYGCACCAAYAGYRCTRTAAA), as described by Baldo et al. [7]. These primers amplify a 513 bp DNA fragment of *Wolbachia* surface protein gene. The amplification reactions were performed in a final volume of 15 μL containing 3 μL of DNA extract, 1.5 μL of 10X PCR reaction buffer, 2 mM of MgCl_2_, 20 picomoles of each primer, 200 mM of each dNTP and 0.3 units of Taq DNA polymerase. The amplification program started with a denaturation step at 94 °C for 3 min followed by 37 amplification cycles; each cycle containing a denaturation step at 94 °C for 30 s, an annealing step at 53 °C for 30 s, and an extension step at 72 °C for one min followed by a final extension at 72 °C for 5 min.

Ten micro-liters of amplified products were analyzed by electrophoresis on 2% agarose gel containing ethidium bromide. Each gel was visualized under UV light and then photographed. All positive samples for *Wolbachia* were subsequently genotyped with five polymorphic genes.

### Multi Locus Sequences Typing (MLST) of *Wolbachia* sp.

For the genotyping, five genes including *gatB, coxA, hcpA, fbpA* and *ftsZ* were analyzed. Each of these genes was amplified with the primers (Table S1) previously described by Baldo et al. [7]. For each gene, PCR reactions were performed as described by Doudoumis et al. [16]. Each PCR reaction was carried out in a final volume of 25 μL containing 2.5 μL of 10X PCR reaction buffer, 2 mM of MgCl_2_, 200 mM of each dNTP, 15 picomoles of each primer, 0.3 units of Taq DNA polymerase (Qiagen 5 U/μL), and 5 μL of DNA extract. The amplification programs consisted of a denaturation step at 94 °C for 5 min, followed by 36 cycles. Each of these cycles contained a denaturation step at 94 °C for 30 s, an annealing step of 30 s at 52 °C for *ftsZ*, 54 °C for *gatB*, 55 °C for *coxA*, 56 °C for *hcpA,* and 58 °C for *fbpA*, and an elongation step at 72 °C for one minute. All reactions were followed by a final extension step at 72 °C for 10 min.

To resolve the PCR products, 10 μL of amplified products were analyzed by electrophoresis on 2% agarose gel containing ethidium bromide. Each gel was visualized under UV light and then photographed. Some positive samples were subsequently purified and sent for sequencing.

### Sequencing of fbpA fragments

To confirm the presence of different bacteria species, the amplified fragments of fbpA from *Wolbachia*, were sequenced. For this sequencing, three samples were randomly selected. For each sample, two amplicons (two replicates) were sequenced. Before the sequencing, PCR products of the selected samples were purified from agarose gel with the GeneJet PCR purification kit (Qiagen). The sequencing reactions were performed by a commercial company (GATC Biotech AG, Germany). Sequences were obtained by long reads from both ends. The complete sequence of fbpA fragment was generated by aligning the long reads from both ends using Codon Code Aligner sequence assembler 3.7.1 software. Each complete sequence of the amplified fbpA fragment was blasted against fbpA sequences of *Wolbachia* strains available in GenBank [35, 50]. The blast was done using the BLASTN 2.6.0+ program of the NCBI (https://blast.ncbi.nlm.nih.gov/blast.cgi).

### Statistical analysis

Statistical analyses were performed using R 3.5.1 [39] Core Team software (R: R Core Team 2018, Vienna, Austria). The frequencies of *S. glossinidius* and *Wolbachia* infections were presented as proportions with 95% confidence intervals (CIs). Fisher’s exact test in R 3.5.1 2018 Core Team software was used to compare *S. glossinidius* and *Wolbachia* infection rates between villages. The difference was considered significant when the *p*-value was lower than 0.05.

### Ethics statement

This study was carried out following the recommendations contained in the Guide for the Care and Use of Animals of the Molecular Parasitology Unit, Department of Biochemistry, University of Dschang.

## Results

### Entomological surveys

During the entomological surveys, 102 pyramidal traps were set up in different villages and a total of 700 tsetse flies, including 155 teneral (22.1%) and 545 non-teneral flies (77.9%), all belonging to *G. f. quanzensis* were collected (Table 1). In different villages, the number of flies varied as follows: 3 at Kabengele, 7 at Tshibila, 10 at Bakwanseke, 16 at Kabala, 203 at Ekubi 2, 222 at Lutendele, and 239 at Bena Mungelu. Due to financial constraints, only 51.4% (360/700) of live tsetse flies were randomly selected, dissected and their midguts preserved for molecular analyses. In all, 360 midguts were subjected to the identification of *S. glossinidius* while, for the same constraints mentioned above, a subset of 184 midguts were subjected to molecular detection of *Wolbachia*.

**Table 1:** Results of entomological surveys.

Villages	No. traps	No. captured flies	ADT	No. teneral flies (%)	No. dissected flies
Bakwanseke	10	10	0.25	0	6
Bena Mungelu	17	239	3.51	70 (29.29)	226
Ekubi 2	14	203	3.63	59 (29.06)	56
Kabala	10	16	0.4	6 (37.5)	14
Kabengele	8	3	0.09	0	2
Lutendele	37	222	1.5	20 (9.01)	49
Tshibila	6	7	0.29	0	7
Total	102	700	1.72	155 (22.14)	360

No.: Number of; ADT: Apparent density per trap per day; (%): Percentage of teneral flies.

### *Sodalis glossinidius* infection rate

**Figure 2:**
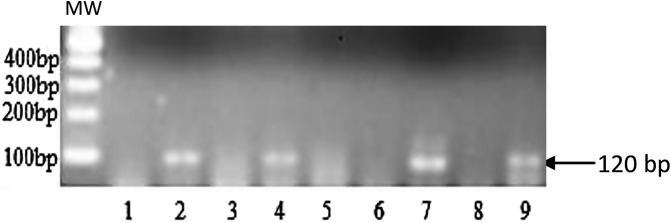
Agarose gel showing the DNA profile resulting from the molecular identification of *S. glossinidius* using pSG2 primers. Lane 1: negative control; lane 9: positive control; lanes 2, 4 and 7: samples positive for *S. glossinidius*; Lane 3, 5, 6 and 8: samples negative for *S. glossinidius*. MW: 100 bp DNA ladder.

Figure 2 shows a representative agarose gel illustrating the 120 bp fragment resulting from the amplification of the PSG gene of *S. glossinidius.* Of the 360 midguts that were investigated for the presence of *S. glossinidius,* 56 were found with *S. glossinidius* infections, yielding an overall infection rate of 15.56%. The highest infection rate was observed at Tshibila (42.86%) followed by Lutendele (22.45%) and Bakwanseke (16.67%). The lowest infection rate of 7.14% was observed at Ekubi 2 and Kabala. The infection rates reported at Kabala, Kabengele, Tshibila and Bakwanseke must be considered with caution because fewer than 15 tsetse flies were analyzed in each of these villages. Despite the variations observed in the infection rates of *S. glossinidius*, no significant difference (*P* = 0.2002) was observed between villages (Table 2). This difference remains not significant (*P* = 0.1018) even with the subset of 184 samples that were subjected to the molecular identification of *Wolbachia* (Table 3).

**Table 2:** Infection rates of *S. glossinidius* according to villages.

Villages	Number of tsetse flies captured	Number of midguts analyzed	Number of midguts with *S. glossinidius* infections (%)	95% CI
Bakwanseke	10	6	1 (16.67)	0.42–64.12
Bena Mungelu	239	226	36 (15.93)	11.73–21.26
Ekubi 2	203	56	4 (7.14)	2.81–16.97
Kabala	16	14	1 (7.14)	1.27–31.47
Kabengele	3	2	0	
Lutendele	222	49	11 (22.45)	13.02–35.88
Tshibila	7	7	3 (42.86)	9.9–81.59
Total	700	360	56 (15.55)	12.18–19.66
*P*-value			0.2002	

(%): *S. glossinidius* infection rate; CI: Confidence interval.

**Table 3:** *Wolbachia* and *S. glossinidius* infection rates according to villages and number of tsetse with co-infections of *Wolbachia* and *S. glossinidius.*

Villages	Number of tsetse flies captured	Number of midguts analyzed	Number of midguts with *Wolbachia* infections (%)	95% CI	Number of midguts with *S. glossinidius* infections (%)	95% CI	Number of midguts with co-infection of *Wolbachia* and *S. glossinidius* (%)	95% CI
Bakwanseke	10	7	5 (71.43)	29.04–96.33	0 (0)		1 (14.28)	0.36–57.87
Bena Mungelu	239	114	111 (97.37)	92.55–99.10	21 (18.42)	12.38–26.52	5 (4.39)	1.89–9.86
Ekubi 2	203	24	21 (87.5)	69–95.65	4 (16.67)	6.68–35.85	1 (4.17)	0.74–20.24
Kabala	16	11	11 (100)	74.12–100	1 (9.09)	1.62–37.73	1 (9.09)	1.62–37.73
Kabengele	3	2	2 (100)	15.81–100	0 (0)		0	
Lutendele	222	20	0 (0)		11 (55)	16.23–37.73	0	
Tshibila	7	6	6 (100)	54.07–100	3 (50)	11.81–88.19	2 (33.33)	4.33–77.72
Total	700	184	156 (84.78)	78.88–89.26	40 (21.74)	16.76–28.84	10 (5.43)	2.99–9.71
*P*-value			0.9967		0.1018		0.0957	

(%): Infection rates; CI: Confidence interval.

### Infection rate of *Wolbachia*

**Figure 3:**
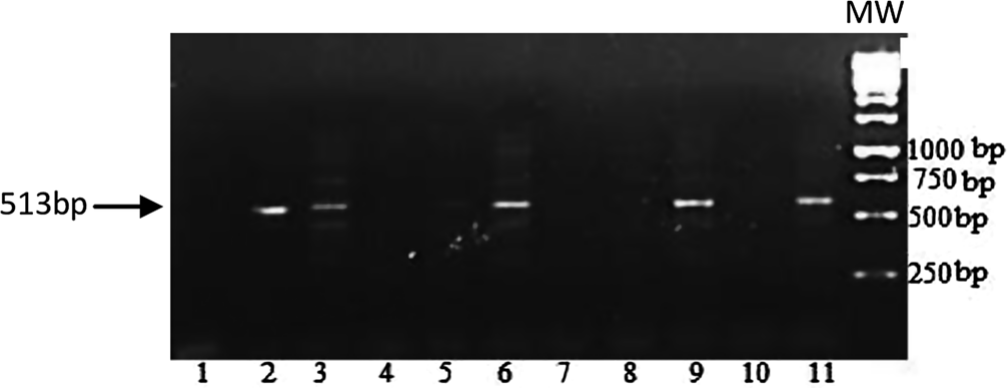
Agarose gel illustrating the DNA profile resulting from the amplification of a DNA fragment of the *Wolbachia* surface protein gene using wsp primers. MW: 1 kb DNA ladder molecular weight marker; lane 1: negative control; lane 11: positive control; Lane 2, 3, 6 and 9: samples positive for *Wolbachia*; lanes 4, 5, 7, 8 and 10: samples negative for *Wolbachia*.

Of the 360 midguts collected during this study, 184 were randomly selected and investigated for the presence of *Wolbachia*. Figure 3 shows a representative agarose gel illustrating the amplification of a *Wolbachia* surface protein DNA fragment of 513 bp, which is specific to *Wolbachia*. Of 184 midguts analyzed in this study, 156 (84.78%) were found to be positive for *Wolbachia* infections (Table 3). No sample from Lutendele revealed midgut infection of *Wolbachia*. All samples from Kabala, Tshibila and Kabengele hosted *Wolbachia*. The infection rate reported in each of these three villages must be considered with caution because the sample size was very low with fewer than 15 midguts analyzed in each of these villages. Despite the variations observed in the *Wolbachia* infection rates, their comparison revealed no significant difference (*P* = 0.9967) between villages (Table 3).

### Genetic characterization of *Wolbachia* sp.

Of the 156 midguts positive for *Wolbachia* infections, a DNA fragment corresponding to the gene coding for the fructose bisphosphate aldolase (fbpA) was amplified in 93 (60%) of them (Table 4). The amplification rate varied according to villages. The highest amplification rate was observed at Bakwanseke (100%) followed by Bena Mungelu (70.27%) (Table 4). To confirm that the amplified fragments of fbpA belonged to *Wolbachia*, six amplicons from three tsetse flies were sequenced. The sequences were subsequently compared to those available in the database. The sequencing revealed fragments ranging from 490 to 498 bp (Table S2). The blast of fbpA sequences with those of other strains available in GenBank revealed more than 90% identity with sequences of *Wolbachia* sp. *wRi*, (complete genome with accession number: CP001391.1), *Wolbachia endosymbiont* of *Drosophila simulans wHa* (complete genome with accession number: CP003884.1), and *Wolbachia* endosymbiont of *Hylaeus variegatus* isolate HVa (complete genome with accession numbers: KP183278.1 and KX843420.1) (Table S2). No difference was observed between the sequences and sizes of fragments originating from the same tsetse fly. However, some slight differences were observed between *Wolbachia* sequences coming from different tsetse flies. For the other selected genes (coxA, ftsZ, gatB and hcpA), no amplification was observed for all the 156 midguts reported with *Wolbachia* infections.

**Table 4:** Amplification rates of the fbpA gene according to the villages.

Villages	Number of midguts analyzed	Number of midguts with amplified fbpA gene (%)	95% CI
Bakwanseke	5	5 (100)	47.83–100
Bena Mungelu	111	78 (70.27)	61.21–77.98
Ekubi 2	21	4 (19.05)	7.67–40
Kabala	11	4 (36.36)	15.17–64.62
Kabengele	2	0 (0)	
Tshibila	6	2 (33.33)	4.33–77.72
Total	156	93 (59.61)	51.77–66.99
*P*-value		0.1062	

(%): Rate of midguts with amplified fbpA gene; CI: Confidence interval.

### Co-infection of *Wolbachia* and *S. glossinidius*

Of the 184 midguts that were simultaneous analyzed for the presence of *Wolbachia* and *S. glossinidius*, 10 (5.43%) revealed co-infections with these two symbionts. Between villages, no significant difference (*P* = 0.0957) was observed in the number of tsetse flies with midguts co-infected by *Wolbachia* and *S. glossinidius* (Table 3).

## Discussion

One of the main challenges that limits our appraisal of the tsetse microbiome is the difficulty encountered in identifying bacterial species that colonize these flies. To contribute to filling the gaps in the understanding of the bacterial flora of certain understudied tsetse species, molecular tools were used to identify *S. glossinidius* and *Wolbachia* in the midgut of *G. f. quanzensis* caught in different regions of the Democratic Republic of Congo.

Although few investigations have been undertaken on *S. glossinidius* in tsetse of the *G. fuscipes* species, the first attempts using standard PCR (amplification of specific gene by conventional PCR), and culture dependent and independent methods failed to identify *S. glossinidius* in *G. f. fuscipes* populations from Uganda [5] and Kenyan [33]. However, other approaches combining standard PCR and qPCR methods that target regions other than the 16S rRNA gene have recently allowed for the confirmation of the presence of *S. glossinidius* in different tsetse species including *G. f. fuscipes* populations from Uganda [1]. Our results showing *S. glossinidius* in *G. f. quanzensis* in the DRC are therefore in agreement with the results of Aksoy et al. [1] and those obtained in other tsetse flies of the *palpalis* and *morsitans* groups [13, 20]. The findings of the present study thus open a framework for investigations aimed at understanding the implications of *S. glossinidius* in the vector competence of *G. f. quanzensis*. Comparing the infection rates of *S. glossinidius* between wild populations of different tsetse species of various regions, our infection rate of 15.6% is similar to 17.5% reported in Zambia for *G. m. morsitans* [13]. This rate is higher than 1.4% and 9.3% reported for *G. pallidipes* in Zambia [13] and *G. p. palpalis* in Liberia [34], but lower than 93.7% and 54.9% reported in Zambia for *G. brevipalpis* [13], and in Cameroon for *G. p. palpalis* [20]. However, in the present study, it is important to point out that *S. glossinidius* was identified in tsetse midguts, while other studies used whole tsetse or parts of the insect such as the abdomen, the thorax and the legs. Although these methodological variations have important implications in the comparison of data resulting from different studies, the results generated by these studies confirm the high heterogeneity of *S. glossinidius* infection rates according to tsetse species [13, 20].

The variations observed in the infection rates (7.14–22.45%) of *S. glossinidius* according to the villages corroborate results obtained in *G. p. palpalis* of two HAT foci of southern Cameroon [20]. They are in agreement with results reported in Kenyan populations of *G. austeni* and *G. pallidipes* where some variations were observed in *S. glossinidius* infection rates according to sampling sites [45]. These variations can be explained by the bacterial density as well as environmental factors (vegetation, humidity, temperature) that differ between villages [45]. The difference in the bacterial diversities could reflect various abiotic factors, such as humidity and wetlands which play a role in environmental exposure of tsetse and its symbiotic microorganisms. In experimental studies where environmental variations are removed and the symbiotic association between tsetse and symbionts is not affected, high vertical transmission of symbionts is observed and high infection rates are expected. The submission of different tsetse populations to constant and specific environmental conditions has probably induced selection of specific tsetse populations characterized by high vertical transmission of *S. glossinidius*. However, in natural conditions where bioclimatic factors vary and have impacts on tsetse biology, the relationship between tsetse and symbionts can be modified. As the survival of symbionts is linked to tsetse biology because of their limited metabolic capacities, each environmental modification affecting the biology of tsetse modifies the molecular interactions between tsetse and symbionts. In such a context, *S. glossinidius* could not easily undergo horizontal transmission and its infection rates could vary with environmental factors.

The identification of *Wolbachia* in *G. f. quanzensis* contrasts with the results of first attempts where no such infection was reported in some tsetse of the *fuscipes* species [10, 16]. However, our results are in line with those of Symula et al. [43] who reported *Wolbachia* in *G*. *f. fuscipes*. The discrepancy between these results can be linked to the variety of tsetse species, the sensitivity of molecular markers and the density of *Wolbachia* in different tsetse species. Indeed, with molecular markers targeting *Wolbachia* surface protein (wsp-PCR), Schneider et al. [42] categorized *Wolbachia* infections into high, intermediate, low and not detectable in *G. m. centralis, G. m. morsitans, G. swynnertoni*, and *G. brevipalpis,* respectively. The same authors applied the PCR-blot technique and significantly enhanced the detection capacity of *Wolbachia* in different tsetse species. Interestingly, this PCR-blot technique made it possible to detect *Wolbachia* in samples of some tsetse species such as *G. swynnertoni* that were previously reported with no infection [42], indicating clearly that *G. swynnertoni* can host *Wolbachia*. These results show some variations in the sensitivity of techniques and molecular markers used to detect symbionts. They also show some variability in the density of *Wolbachia* according to tsetse species. These factors have impacts on the *Wolbachia* infection rates and highlight the need to develop sensitive tools for a real evaluation of *Wolbachia* infection in different tsetse species.

Our infection rate of 84.8% is lower than the 100% reported in *G. austeni* [45]. This discrepancy can be linked to tsetse species because each of them is characterized by specific molecular interactions with its symbiotic microorganisms. These interactions have impacts on the density of *Wolbachia* and subsequently, on its infection rate. The infection rate of 84.8% reported here does not reflect the real prevalence of *Wolbachia* in *G. f. quanzensis* because only midguts were investigated. With such focused investigations, the real prevalence of *Wolbachia* was probably underestimated because *Wolbachia* can be found in other parts such as the head, thorax, abdomen and legs [45]. Some of the 15.2% midguts reported with no infection could come from insects that harbor *Wolbachia* in other tissues not considered in this study. Some of these midguts may host low *Wolbachia* density below the detection threshold of the markers used. The heterogeneity observed in *Wolbachia* infection rates according to villages is in agreement with the observations of Cheng et al. [10], reporting significant variability in wild tsetse populations. In field conditions, bioclimatic factors affect the symbiotic association between tsetse and its symbionts, and consequently the infection rates of symbiotic microorganisms [16, 40].

The midgut infection by *Wolbachia* contrasts with previous observations reporting the main localization of *Wolbachia* in tsetse fly ovaries. Our results are in agreement with those of Wamwiri et al. [45] who used similar approaches to detect *Wolbachia* in the head, thorax, abdomen and legs of *G. austeni*. Although Wamwiri et al. [45] found *Wolbachia* in the abdomen (probably containing midgut and ovary) of the tsetse fly, the results of their study and those generated here show that *Wolbachia* is not just confined to gonads, but could colonize other tsetse tissues. The presence of *Wolbachia* in other tissues could result from specific *Wolbachia* strains probably with large tissue tropism in some tsetse species as reported in other insects [14]. For instance, *Wolbachia* has been detected in ovary and non-gonadal tissues of *Culex quinquefasciatus,* with high density in ovaries compared to non-gonadal tissues [19]. As in *C. quinquefasciatus*, the density of *Wolbachia* could also vary according to tsetse tissues. If *Wolbachia* density is high in ovaries compared to other tissues like tsetse midguts, we can therefore understand why these bacteria have never been detected in such tissues. This hypothesis is strengthened by the observations of Wamwiri et al. [45] who found differences in the intensity of PCR products of *Wolbachia* specific fragments between Kenyan and the South African tsetse populations. The *Wolbachia* strains found in the tsetse midgut may have specific characters like large tissue tropism and invasion capacity that are probably different from those of strains previously studied. Such tissue tropism has already been reported in other tsetse species such as *G. auteni* [10]. Genetic investigations on these strains could help to decrypt and understand the differences observed. Moreover, in the current elimination context of Human African trypanosomiases, the identification of *Wolbachia* in tsetse midgut opens a framework for investigations intended to develop paratransgenic approaches for vector-control. If *Wolbachia* prevent trypanosome infections, the presence of specific strains in tsetse midgut could open new perspectives for vector control where trypanosomes establishment could be prevented. Although the prevention mechanism is not yet understood, recent investigations highlight a negative correlation between *Wolbachia* and the presence of trypanosomes [2, 5]. Similar observations have already been described in other vector-borne diseases. In mosquitoes for instance, *Wolbachia* has been reported to confer resistance against *Plasmodium* and dengue virus through the stimulating the expression of certain genes related to the host’s immune response [8, 30]. Assuming the presence of *Wolbachia* in tsetse midgut, we can speculate that its direct contact with trypanosomes could prevent the establishment of this parasite, possibly due to interaction with the host and a subsequent impact on the host immune response, rather than direct contact with the trypanosome. Decrypting *Wolbachia-*tsetse-trypanosome interactions for the final goal of understanding how *Wolbachia* could prevent trypanosome establishment requires additional investigations.

Of the five genes (*fbpA, hcpA, gatB, coxA* and *ftsZ*) selected for MLST, only *fbpA* was amplified in about 60% of *Wolbachia* positive samples. The amplification, sequencing and comparison of fbpA sequences with those available in the database showed that the amplified sequences belonged to *Wolbachia*, thus confirming its presence in the tsetse midgut. For the other genes, no amplification was observed. These results could be explained by the low titer of *Wolbachia* that could be below the detection threshold of “standard” PCR (amplification of a specific gene by conventional PCR). In fact, Schneider et al. [42] have shown some *Wolbachia* strains escaping the “standard” PCR methods by hiding as low titer below the detection threshold. Applying a highly sensitive PCR-blot technique to the same samples, these authors observed a high prevalence of *Wolbachia* infections, even in tsetse species initially found with no infection. In addition, *Wolbachia* genomes can undergo frequent rearrangements and rapid evolution due to the high number of transposable elements and repeat regions that provide sites for recombination [9, 22, 31]. As reported in other bacteria [9, 17, 38], these permanent genetic modifications could lead to mutations at the primer binding sites.

This study showed that only 5.43% of *G. f. quanzensis* midguts were co-infected by *Wolbachia* and *S. glossinidius*. This result indicates that the midguts of natural populations of *G. f. quanzensis* are rarely coinfected by *Wolbachia* and *S. glossinidius.* This low midgut co-infection of *Wolbachia* and *S. glossinidius* can be linked to the differences observed in the biological effects of these bacteria. The presence of trypanosomes was reported to be negatively correlated (*r* = −0.176) with *Wolbachia* infection, suggesting that infection by *Wolbachia* may prevent trypanosome infections [2, 5]. On the contrary, the presence of *S. glossinidius* seems to favor trypanosome infections [20]. These observations indicate that some antagonistic actions resulting from the biological effects of *Wolbachia* and *S. glossinidius* may occur in tsetse midgut during trypanosome infections. They also indicate difficult co-association between *S. glossinidius* and *Wolbachia* in the tsetse midgut. The low prevalence of co-infections between *S. glossinidius* and *Wolbachia* is probably underestimated because only tsetse midguts were analyzed. Since *S. glossinidius* and *Wolbachia* can be found in other tissues not analyzed here, investigations of such tissues could increase the co-infection rates of these bacteria. A better understanding of the association between these bacteria by continuing investigations on the infection rates of both *Wolbachia* and *S. glossinidius* could help to develop new vector control strategies. For example, sustained elimination of HAT could be achieved over 25 years when *Wolbachia* colonization minimally impacted fecundity or mortality, and when the probability of recombinant *Sodalis* vertical transmission exceeded 99.9% [25].

## Conclusion

This study revealed *Wolbachia* and *S. glossinidius* in *G. f. quanzensis* for the first time. The infection rates of these bacteria vary between villages due to climatic and environmental factors. Our results showed that few tsetse flies harbor midgut co-infections of *Wolbachia* and *S. glossinidius*. The data generated in this study have improved our knowledge of the bacterial flora of *G. f. quanzensis* and opened a framework for investigations aimed at understanding the implication of these symbiotic microorganisms in the vectorial competence of *G. f. quanzensis*. In the elimination context where control strategies have to be improved, more data on the simultaneous presence of *Wolbachia* and *S. glossinidius* could help to develop new approaches for vector control.

## Supplementary material

Supplementary materials are available at https://www.parasite-journal.org/10.1051/parasite/2019005/olm.
