# Supplementary material for: Molecular identification of Wolbachia and Sodalis glossinidius in the midgut of Glossina fuscipes quanzensis from the Democratic Republic of Congo
Source: Parasite. 2019 Feb 7;26:5. doi: 10.1051/parasite/2019005 (PMC6366345; doi:10.1051/parasite/2019005)
Supplement: Supplementary file 1 [file parasite-26-5-olm.pdf]

Supplementary tables

Table S1: Primers used for Multi Locus Sequences Typing of *Wolbachia*

Genes	Designation	Primer sequences 5'-3'	References
gatB	gatB_F1	GAKTTAAAYCGYGCAGGBGTT	[6]
	gatB_R1	TGGYAAYTCRGGYAAAGATGA	
coxA	coxA_F1	TTGGRGCRATYAACTTTATAG	[6]
	coxA_R1	CTAAAGACTTTKACRCCAGT	
hcpA	hcpA_F1	GAAATARCAGTTGCTGCAAA	[6]
	hcpA_R1	GAAAGTYRAGCAAGYTCTG	
ftsZ	ftsZ_F1	ATYATGGARCATATAAARGATAG	[6]
	ftsZ_R1	TCRAGYAATGGATTRGATAT	
fbpA	fbpA_F1	GCTGCTCCRCTTGGYWTGAT	[6]
	fbpA_R1	CCRCCAGARAAAAYYACTATTC	

Table S2: Blast results of fbpA sequences with those of *Wolbachia* strains in the database

***Wolbachia* species**	**Size of fragments (bp)**	**Query coverage**	**E-value**	**Identity**	**Accession number**
Wolbachia sp. *wRi*. Complete genome	490<br>498	100%<br>99%	0.0<br>0.0	90%<br>89%	CP001391.1
Wolbachia endosymbiont of Drosophila simulans wHa. Complete genome	490<br>498	100%<br>99%	0.0<br>0.0	90%<br>89%	CP003884.1
Wolbachia endosymbiont of Hylaeus variegatus isolate HVa fructose-bisphosphate aldolase (fbpA) gene	490<br>498	96%<br>94%	0.0 7e-179	91%<br>90%	KP183278.1
Wolbachia pipientis voucher wol_sc_02621 fructose-bisphosphate aldolase (fbpA) gene	490<br>498	93%<br>91%	0.0 3e-177	91%<br>90%	KX843420.1
